# Transcriptome Analysis Reveals the Induction of Apoptosis-Related Genes by a Monoclonal Antibody against a New Epitope of CD99 on T-Acute Lymphoblastic Leukemia

**DOI:** 10.3390/antib13020042

**Published:** 2024-05-17

**Authors:** Nuchjira Takheaw, Kamonporn Kotemul, Ratthakorn Chaiwut, Supansa Pata, Witida Laopajon, Kuntalee Rangnoi, Montarop Yamabhai, Watchara Kasinrerk

**Affiliations:** 1 Division of Clinical Immunology, Department of Medical Technology, Faculty of Associated Medical Sciences, Chiang Mai University, Chiang Mai 50200, Thailand; nuchjira.t@cmu.ac.th (N.T.); kamonporn_kote@cmu.ac.th (K.K.); witida.l@cmu.ac.th (W.L.); 2 Biomedical Technology Research Center, National Center for Genetic Engineering and Biotechnology, National Science and Technology Development Agency at the Faculty of Associated Medical Sciences, Chiang Mai University, Chiang Mai 50200, Thailand; 3 Molecular Biotechnology Laboratory, School of Biotechnology, Institute of Agricultural Technology, Suranaree University of Technology, Nakhon Ratchasima 30000, Thailandmontarop@g.sut.ac.th (M.Y.)

**Keywords:** anti-CD99 monoclonal antibody, CD99 epitope, T-cell acute lymphoblastic leukemia, apoptosis, antibody therapy

## Abstract

CD99 was demonstrated to be a potential target for antibody therapy on T-acute lymphoblastic leukemia (T-ALL). The ligation of CD99 by certain monoclonal antibodies (mAbs) induced T-ALL apoptosis. However, the molecular basis contributing to the apoptosis of T-ALL upon anti-CD99 mAb engagement remains elusive. In this study, using our generated anti-CD99 mAb clone MT99/3 (mAb MT99/3), mAb MT99/3 engagement strongly induced apoptosis of T-ALL cell lines, but not in non-malignant peripheral blood cells. By transcriptome analysis, upon mAb MT99/3 ligation, 13 apoptosis-related genes, including FOS, TNF, FASLG, BCL2A1, JUNB, SOCS1, IL27RA, PTPN6, PDGFA, NR4A1, SGK1, LPAR5 and LTB, were significantly upregulated. The epitope of CD99 recognized by mAb MT99/3 was then identified as the VDGENDDPRPP at residues 60–70 of CD99, which has never been reported. To the best of our knowledge, this is the first transcriptome data conducted in T-ALL with anti-CD99 mAb engagement. These findings provide new insights into CD99 implicated in the apoptosis of T-ALL. The identification of a new epitope and apoptosis-related genes that relate to the induction of apoptosis by mAb MT99/3 may serve as a new therapeutic target for T-ALL. The anti-CD99 mAb clone MT99/3 might be a candidate for further development of a therapeutic antibody for T-ALL therapy.

## 1. Introduction

CD99 is a tumor-associated antigen that showed strong expression levels in T-acute lymphoblastic leukemia (T-ALL) but low expression levels in normal cells [1,2,3]. Thus, CD99 was suggested to be a marker for assessing minimal residual disease (MRD) in T-ALL in 2004 [2]. However, over the past 25 years, several clones of anti-CD99 mAb were generated and used as a tool to investigate the roles of CD99 in T cell regulation [4,5]. The application of anti-CD99 mAbs as a therapeutic antibody for T-ALL, nevertheless, has not been focused on.

Currently, several antibodies have demonstrated their ability to significantly improve the clinical outcome of patients, particularly in several cancer types [6]. Around 100 monoclonal antibodies (mAbs) have been approved by the Food and Drug Administration (FDA) for the treatment of various solid tumors and hematological malignancies [7]. Unfortunately, antibody drugs for the treatment of T-ALL are still limited. One of the major roadblocks is that most tumor antigens for malignant T cells are often also expressed on non-malignant T cells [8]. The shared expression of tumor antigens results in the killing of non-malignant and malignant T cells by antibodies and causes immunosuppression [8,9]. Consequently, the discovery of mAbs against targeted molecules that exert a direct killing effect only on malignant T cells has been the focus. Currently, CD99 has been demonstrated to be a potential target for antibody therapy on T-ALL with less toxicity to normal blood cells [10,11]. It has been reported that targeting CD99 by certain clones of anti-CD99 mAb induced cell apoptosis of malignant T cells but not in non-malignant T cells [12,13,14]. However, there remains a lack of knowledge on which epitope on the CD99 molecule contributes to the apoptosis of T-ALL upon anti-CD99 mAb engagement. Moreover, the molecular basis upon anti-CD99 mAb engagement remains elusive.

The anti-CD99 mAb clone MT99/3 (mAb MT99/3) was generated in our research center [15]. This mAb has been demonstrated to induce homotypic adhesion of Jurkat T-ALL cells [15]. In the present study, we employed mAb MT99/3 to determine whether this mAb has a potential therapeutic property for T-ALL treatment. We demonstrated that mAb MT99/3 could induce apoptosis in T-ALL cell lines, but not in non-malignant peripheral blood cells. To facilitate the identification of apoptosis-related genes and provide a comprehensive understanding of molecular mechanisms underlying CD99 ligation, the transcriptome analysis upon mAb MT99/3 engagement in a T-ALL cell line was performed. The apoptosis-related genes, which were significantly upregulated, were revealed. Additionally, the death signals induced by anti-CD99 antibodies were diverse depending on the binding with different epitopes on the CD99 molecule. We identified the epitope recognized by mAb MT99/3 and found that this mAb binds to the epitope of CD99 that has never been reported. The identification of a new epitope and apoptosis-related genes that relate to the induction of apoptosis by mAb MT99/3 may serve as a new therapeutic target for T-ALL. The anti-CD99 mAb clone MT99/3 might be a candidate for the development of a therapeutic antibody for the treatment of T-ALL patients.

## 2. Materials and Methods

### 2.1. Cells, Cell Lines and Antibodies

The T-ALL cell lines, Jurkat E6.1 and MOLT-4, and the T-lymphoblastic lymphoma (T-LBL) cell line, SUP-T1, were obtained from the American Type Culture Collection (ATCC, Manassas, VA, USA). These cells were cultured in RPMI 1640 (Gibco, Grand Island, NY, USA) supplemented with 10% heat-inactivated fetal bovine serum (FBS, Life Technologies, Paisley, UK) (10% FBS-RPMI) at 37 °C in a humidified 5% CO_2_ incubator.

Human PBMCs were isolated from buffy coats of healthy individuals obtained from the Reginal Blood Center X, Thai Red Cross Society, Chiang Mai, Thailand or heparinized whole blood of healthy donors, using standard Ficoll-Hypaque density gradient centrifugation. 

The anti-human CD99 mAb clone MT99/3 (mouse IgG2a) was produced from a hybridoma clone generated in our laboratory [15]. Anti-dengue viral protein mAb clone 4G2 (mouse IgG2a, isotype-matched control mAb) was produced from a hybridoma clone obtained from Dr. Prida Malasit (Division of Medical Molecular Biology, Faculty of Medicine Siriraj Hospital, Mahidol University, Bangkok, Thailand).

### 2.2. Cell Apoptosis Assay

Cell lines or PBMCs (1 × 10^5^/100 µL) were pre-incubated with mAb MT99/3 at a final concentration of 20 µg/mL or isotype-matched control mAb 4G2 or medium (No Ab) for 30 min at room temperature. The treated cells were centrifuged and the excess mAbs were removed. The secondary antibody crosslinker goat anti-mouse IgG antibody (#115-005-072 Jackson ImmunoResearch Laboratories, West Grove, PA, USA) at 5 µg/mL, 150 µL were added into the treated cells and incubated for 30 min at room temperature. After that, the activated cells were incubated at 37 °C in a humidified 5% CO_2_ incubator for 0 min, 90 min and 180 min. Treated cells were harvested and stained with apoptosis markers Annexin V-FITC (BioLegend, San Diego, CA, USA) and 7-AAD solution (BioLegend). Cell apoptosis was determined by a BD Accuri C6 Plus flow cytometer (BD Biosciences, San Jose, CA, USA) and analyzed using FlowJo software V10.6.2.

### 2.3. RNA Extraction

Jurkat E6.1 T cell line was pre-incubated with 20 µg/mL of tested mAb MT99/3 or isotype-matched control mAb 4G2 for 30 min at room temperature. The excess mAbs were removed, followed by incubation with goat anti-mouse IgG antibody at 150 µL of 5 µg/mL for 30 min at room temperature. The activated cells were incubated at 37 °C in a humidified 5% CO_2_ incubator for 90 min. Treated cells were harvested and separated into two experiments which were the analysis of cell apoptosis by flow cytometry and RNA extraction for transcriptome sequencing.

Each treated condition was performed in three independent experiments. For the analysis of cell apoptosis by flow cytometry, treated cells were stained with apoptosis markers (Annexin V-FITC and 7-AAD solution). Cell apoptosis was determined by a BD Accuri C6 Plus flow cytometer. For RNA extraction, the total RNA of each condition was extracted from the treated cells using the Illustra RNAspin Mini RNA Isolation Kit (GE Healthcare, Buckinghamshire, UK). The amounts of total RNA were quantified by NanoDrop, and the quality of RNA was analyzed by Agilent Bioanalyzer 2100 (Agilent Technologies, Palo Alto, CA, USA).

### 2.4. mRNA Library Constructing and Sequencing

The mRNA library construction and sequencing were carried out by Vishuo Biomedical, Bangkok, Thailand. An amount of 1 µg of total RNA was used for library preparation as follows. Oligo(dT) beads were used for the poly(A) mRNA isolation. The mRNA fragmentation was carried out using divalent cations and high temperatures. Random Primers were used for priming. First-strand cDNA and second-strand cDNA were synthesized. The purified double-stranded cDNA was then treated to repair both ends. A dA-tailing was added, followed by a T-A ligation to add adaptors to both ends. After that, DNA Clean Beads (Vazyme, Nanjing, China) were used for size selection of adaptor-ligated DNA. Each sample was then amplified by PCR using P5 and P7 primers, and the PCR products were validated. Libraries with different indexes were multiplexed and loaded on an Illumina Novaseq instrument (Illumina, San Diego, CA, USA) for sequencing using a 2 × 150 paired-end (PE) configuration according to the manufacturer’s instructions.

### 2.5. RNA-Sequencing Data Analysis

During sequencing, quality concerns were considered. A small number of target sequences might be read into adapter sequences, and bases toward the 3′ end might have low quality due to the lengthy sequencing cycles. To eliminate the negative effect of these technical issues, low-quality reads, adapter sequences and contaminations were filtered out before data analysis. For filtering, the raw data of six samples were processed by Cutadapt (version 1.9.1) to remove the adapter sequences, the 5′ or 3′-end bases that contain N’s or of quality values below 20, bases with an average quality score below 20 using the sliding window of 4 bp and reads that were less than 75 bp long after trimming. The Q20, Q30 and GC content of the clean data were calculated. All the downstream analyses were based on high-quality cleaned data.

For the determination of differentially expressed genes, RNA differential expression between isotype-matched control mAb 4G2 and mAb MT99/3 treated groups was analyzed using DESeq2 software V1.6.3. We defined the genes with significant differential expression according to the criteria of fold change greater than 2 and Qvalue (fdr, padj) of less than 0.05.

For the Kyoto Encyclopedia of Genes and Genomes (KEGG) enrichment analysis, genes involved in the same biological functions were classified into pathways. The Kyoto Encyclopedia of Genes and Genomes is a pathway-related database resource for facilitating the determination of the differentially expressed genes involved in the most important biochemical metabolic pathways and signal transduction pathways [16,17]. We used in-house scripts to enrich significant differential expression genes in KEGG pathways.

### 2.6. Real-Time Quantitative RT-PCR

Jurkat E6.1 T cell line was treated with mAb MT99/3 or isotype-matched control mAb 4G2 and RNA was extracted as described above. Each treated condition was performed in three independent experiments. In total, 100 ng of total RNA was reversed to cDNA at a final volume of 20 µL by RevertAid First Strand cDNA Synthesis Kit (Thermo Scientific, Waltham, MA, USA). A 20 μL real-time RT-PCR reaction consisted of 2 μL cDNA, 10 μL SYBR mixture of THUNDERBIRD^TM^ SYBR^®®^ qPCR Mix (Toyobo, Osaka, Japan), 3 μL forward primer (Fw) and reverse primer (Rv) at a final concentration of 0.3 µM and 2 μL nuclease-free water. The reference gene (GAPDH) was used in the same PCR run with tested genes. Primers used were synthesized by Bio Basic Inc. (Toronto, ON, Canada), as listed below:

FOS—Fw: 5′-CTTACTACCACTCACCCGCA-3′
FOS—Rv: 5′-AGTGACCGTGGGAATGAAGT-3′
TNF—Fw: 5′-TGCACTTTGGAGTGATCGGC-3′
TNF—Rv: 5′-ACTCGGGGTTCGAGAAGATG-3′
FASLG—Fw: 5′-TACCAGCCAGATGCACACAG-3′
FASLG—Rv: 5′-GGCATGGACCTTGAGTTGGA-3′
BCL2A1—Fw: 5′-GATAAGGCAAAACGGAGGCTG-3′
BCL2A1—Rv: 5′-ATGGAGTGTCCTTTCTGGTCAA-3′
JUNB—Fw: 5′-AACAGCCCTTCTACCACGAC-3′
JUNB—Rv: 5′-CAGGCTCGGTTTCAGGAGTT-3′
SOCS1—Fw: 5′-AGCTGCACGGCTCCTG-3′
SOCS1—Rv: 5′-TGTGGAGACTGCATTGTCGG-3′
IL27RA—Fw: 5′-ACTTGAACTGCTCGTGGGAG-3′
IL27RA—Rv: 5′-CCTTAGTGCCCCAGACAAGG-3′
PTPN6—Fw: 5′-ACCTCAAGTACCCGCTGAAC-3′
PTPN6—Rv: 5′-GGCTCTCACGCACAAGAAAC-3′
PDGFA—Fw: 5′-TACCTCGCCCATGTTCTGGC-3′
PDGFA—Rv: 5′-TCCCTACGGAGTCTATCTCCAGG-3′
NR4A1—Fw: 5′-CCACATTGTTGCCAAGACCTG-3′
NR4A1—Rv: 5′-CTGGTGTCCCATATTGGGCTT-3′
SGK1—Fw: 5′-GGCATGGTGGCAATTCTCATCG-3′
SGK1—Rv: 5′-AGGTTGATTTGCTGAGAAGGACT-3′
LPAR5—Fw: 5′-CGCAGAGCAACACGGA-3′
LPAR5—Rv: 5′-GGTCATGGGAATGTGGGCTA-3′
LTB—Fw: 5′-CAGCAAGGACTGGGGTTTC-3′
LTB—Rv: 5′-GCCTGTTCCTTCGTCGTCT-3′
GAPDH—Fw: 5′-GCAAATTCCATGGCACCGT-3′
GAPDH—Rv: 5′-TCGCCCCACTTGATTTTGG-3′

The reaction was performed using a QIAquant 96 5plex instrument (QIAGEN, Hilden, Germany). Thermal cycling was carried out following the instructions below: 95 °C for 1 min, 40 cycles at 95 °C for 15 s, 55 °C for 15 s and 72 °C for 30 s. The GAPDH housekeeping gene was used as the gene to normalize the gene expression levels of the tested genes in each treated condition. The relative gene expression level was analyzed using the 2^−ΔΔCT^ method.

### 2.7. Epitope Mapping via Phage Display Random Peptide Library

The phage peptides against anti-CD99 mAb clone MT99/3 were selected from a 12-mers phage display random peptide library (SUT12) by a bio-panning method, as previously described [18,19]. Briefly, a gradually reduced amount of mAb MT99/3, ranging between 10, 5 and 2 µg for each consecutive round of affinity selection, was used. After three rounds of bio-panning, phage ELISA was performed to identify binding activity against mAb MT99/3. The twelve individual phage clones were randomly picked and amplified, as previously reported [20]. Phagemid DNA was prepared from the 11 positive phage clones for nucleotide sequencing by automated DNA sequencing services using the -96gII primer (5′-CCC TCA TAG TTA GCG TAA CG-3′). SnapGene software V1.1.3 was used to analyze amino acid sequences. 

### 2.8. Epitope Mapping via Overlapping Peptide Libraries

Twelve sets of CD99 overlapping peptide libraries were synthesized by GenScript, Nanjing, China. The CD99 overlapping peptides were tagged with biotin-6-aminohexanoic acid (Ahx) at N-terminus. Avidin (Sigma-Aldrich, St. Louis, MO, USA) was coated onto a 96-well ELISA plate (Corning Incorporated, Kennebunk, ME, USA) (10 µg/mL, 50 µL/well). After removing avidin, the plate was blocked with 100 µL of 2% BSA in phosphate buffer saline (PBS) at 37 °C for 1 h and then washed out. Peptides were diluted to 2 µg/mL. A volume of 50 µL of the diluted peptides was added to each well and incubated at 37 °C for 1 h. The mAb MT99/3 or isotype-matched control mAb 4G2 was diluted to 5 µg/mL. Then, a volume of 50 µL of the diluted antibodies or buffer without antibodies was added to each peptide and incubated at 37 °C for 1 h. After excess antibodies were washed out, 50 µL of horseradish peroxidase-conjugated rabbit anti-mouse immunoglobulin Abs (Dako, Glostrup, Denmark) at a dilution of 1:2000 was added into each well and incubated at 37 °C for 1 h. After that, the plate was washed and 3,3′,5,5′-tetramethylbenzidine (TMB) substrate (Invitrogen, Grand Island, NY, USA) was added. The reaction was stopped using 1M HCl and the absorbance was measured at 450 nm.

### 2.9. Statistical Analysis

All statistical analyses of apoptosis assay and RT-qPCR were performed using GraphPad Prism version 9.3.1 (GraphPad Software, Boston, MA, USA). Data were expressed as mean ± SD. The two-way ANOVA and Mann–Whitney U test were used for comparison. *p* < 0.05 was considered statistically significant.

## 3. Results

### 3.1. mAb MT99/3 Strongly Induces Apoptosis in T-Acute Lymphoblastic Leukemia Cell Lines

Anti-CD99 mAb clone MT99/3 (mAb MT99/3) was generated in our research center. To confirm whether mAb MT99/3 could recognize CD99 on malignant T cell lines, including Jurkat E6.1, MOLT-4 and SUP-T1, as well as human PBMCs, indirect immunofluorescence staining was performed. As expected, the flow cytometric analysis showed that mAb MT99/3 exhibited positive reactivity with all tested cells (Supplementary Figure S1). 

**Figure 1:**
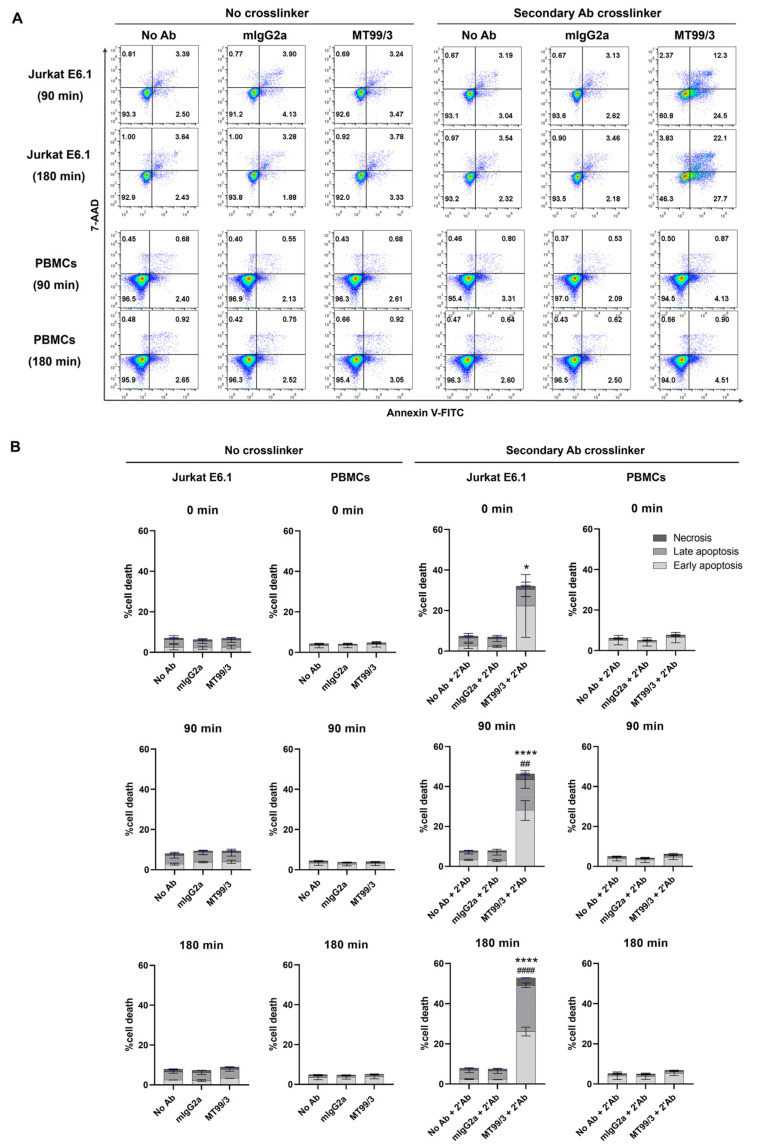
The apoptosis induced by mAb MT99/3 on Jurkat cell line compared with normal PBMCs at 0, 90 and 180 min in 37 °C incubator. Jurkat E6.1 cells and PBMCs were treated with mAb MT99/3, mIgG2a (isotype-matched control mAb 4G2), and no Ab (medium control) in the presence or absence of a secondary antibody crosslinker. Treated cells were incubated at room temperature after adding a secondary antibody crosslinker for 30 min before being incubated at 37 °C incubator for 0, 90 and 180 min. Jurkat E6.1 cells (*n* = 2) and PBMCs (*n* = 5) were performed in two independent experiments. Annexin-V-FITC and 7-AAD were used to indicate apoptosis cells and analyzed using flow cytometric analysis. (**A**) The representative flow cytometric data of the Jurkat E6.1 and PBMCs treatment at 90 and 180 min are shown. (**B**) The bar graphs express the percentages of cell death in early apoptosis (Annexin V^+^7-AAD^−^, light gray), late apoptosis (Annexin V^+^7-AAD^+^, gray) and necrosis (Annexin V^−^7-AAD^+^, dark gray) as mean ± SD. Two-way ANOVA followed by Tukey’s test was used for early apoptosis induced by mAb MT99/3 versus mIgG2a, * *p* < 0.05. **** *p* < 0.0001. For late apoptosis induced by mAb MT99/3 versus mIgG2a, ## *p* < 0.01. #### *p* < 0.0001.

As previously reported, certain clones of anti-CD99 mAb induced apoptosis in malignant T-ALL cells but not non-malignant PBMCs [12,13,14]. We, thus, investigated the direct killing effect of mAb MT99/3 in Jurkat E6.1 T-ALL cell line compared with human PBMCs. By apoptosis assay, Jurkat E6.1 cells and PBMCs (*n* = 5) were treated with mAb MT99/3, isotype-matched control (mAb 4G2) or without an antibody (medium control) in the presence or absence of a secondary antibody crosslinker. The results showed that mAb MT99/3 with a secondary antibody crosslinker significantly induced apoptosis in Jurkat E6.1 cells in a time-dependent manner (Figure 1). Interestingly, this mAb did not induce apoptosis of PBMCs (Figure 1). In the absence of a secondary antibody crosslinker, however, mAb MT99/3 did not induce cell apoptosis in any tested cell (Figure 1). The results demonstrated that mAb MT99/3 could induce T-ALL cell apoptosis, and the clustering of CD99 molecules was required to activate apoptosis signaling. This apoptosis induction occurred only in Jurkat E6.1 T-ALL cells, but not in normal cells.

**Figure 2:**
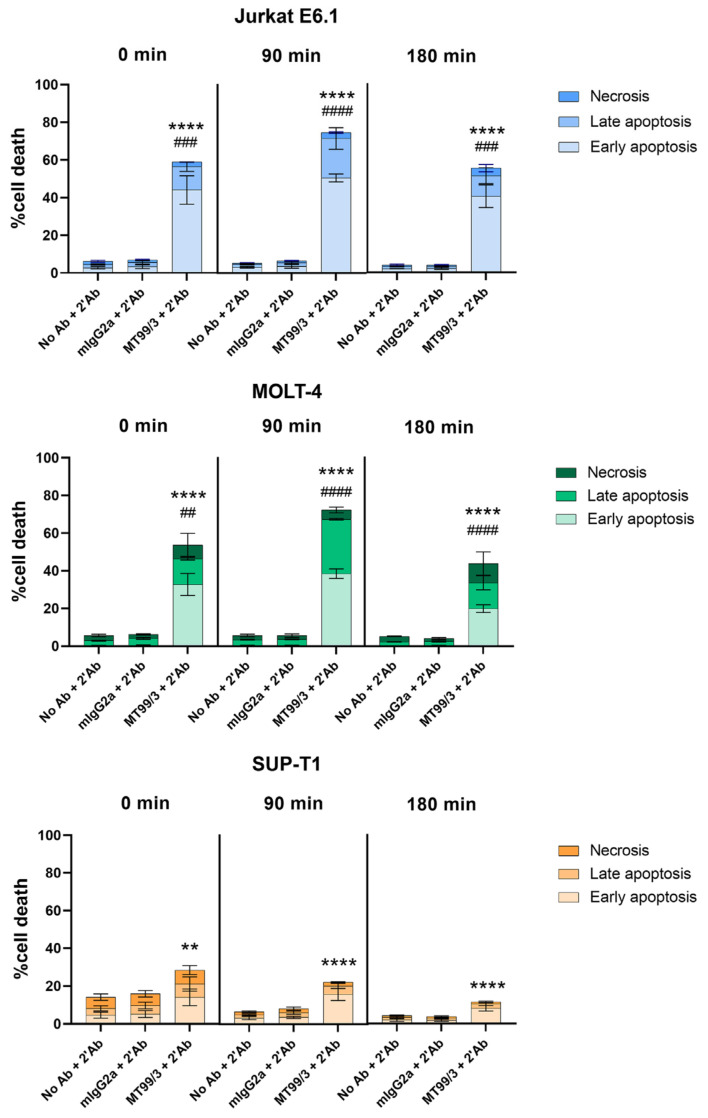
The apoptosis induced by mAbs MT99/3 on malignant T cells. Jurkat E6.1, MOLT-4, SUP-T1 cells were treated with mAb MT99/3, mIgG2a (isotype-matched control mAb 4G2) and no Ab (medium control) in the presence of secondary antibody crosslinker. Treated cells were incubated at room temperature after adding a secondary antibody crosslinker for 30 min before being incubated at 37 °C incubator for 0, 90 and 180 min. The treated cells were stained with Annex-in-V-FITC and 7-AAD. The percentages of cell death in early apoptosis (Annexin V^+^7-AAD^−^), late apoptosis (Annexin V^+^7-AAD^+^) and necrosis (Annexin V^−^7-AAD^+^) were analyzed by flow cytometry. The bar graphs express the mean ± SD of three independent experiments. Two-way ANOVA followed by Tukey’s test was used for early apoptosis induced by mAbs MT99/3 versus mIgG2a, ** *p* < 0.01. **** *p* < 0.0001. For late apoptosis induced by mAbs MT99/3 versus mIgG2a, ## *p* < 0.01. ### *p* < 0.001. #### *p* < 0.0001.

Furthermore, cell apoptosis induced by mAb MT99/3 in another two T cell lines, MOLT-4 and SUP-T1 cells, was determined. Jurkat E6.1 cells line was used as a positive control. The results demonstrated that mAb MT99/3 with a secondary antibody crosslinker could significantly induce cell apoptosis in all T cell lines (Figure 2). Notably, the cell apoptosis induced by mAb MT99/3 was strongest in Jurkat E6.1 and MOLT-4 T-ALL cell lines (Figure 2).

### 3.2. mAb MT99/3-Induced Differential Gene Expression

To identify the molecular mechanism of CD99 involved in the apoptosis of malignant T cells at the transcriptome level, the transcriptome analysis was carried out. Jurkat E6.1 cells were treated with mAb MT99/3 or isotype-matched control mAb 4G2 in the presence of a secondary antibody crosslinker. Cells were then harvested and separated into two experiments, first to confirm the induction of apoptosis had occurred. Second, RNA was extracted for transcriptome analysis. In each treated condition, three independent experiments were performed.

The mAb MT99/3 could induce cell apoptosis of Jurkat E6.1 cells in all experiments performed (Supplementary Figure S2). RNA extracted from all conditions was measured A260/280 ratio and was >1.8, indicating pure RNA with low protein contamination. The RNA extracted was then used to generate a library. For analysis of the quality of RNA, an Agilent 2100 Bioanalyzer was used to measure the RNA integrity. An RNA Integrity Number (RIN) of 10 is indicative of the best-quality RNA. The RIN of >8 was observed in our RNA preparation, and this was acceptable for transcriptome analysis (Supplementary Figure S2).

To identify which genes were regulated by mAb MT99/3 engagement, transcriptome analyses were carried out on three independent samples. Illumina Novaseq was used for sequencing. Low-quality reads, adaptor sequences and contaminations were filtered out before data analysis. The clean reads with high quality were used for further analyses. The numbers of clean reads are shown in Supplementary Table S1, in which isotype-matched control mAb-treated groups were C1, C2 and C3, and mAb MT99/3-treated groups were T1, T2 and T3. The Q30 proportion of all six samples exceeded 92%. This indicated that the quality of the clean reads was high. The results obtained met the requirement for further bioinformatics analyses.

**Figure 3:**
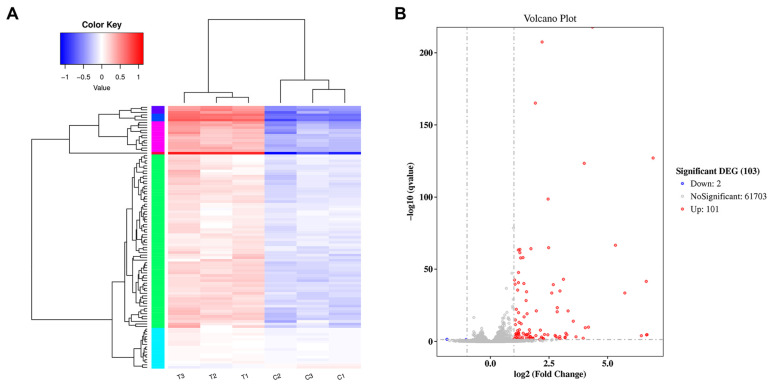
Determination of differentially expressed genes. (**A**) Cluster analysis of differentially expressed genes Log_10_ (FPKM + 1) values were used for clustering. FPKM stands for fragments per kilobase of exon model per million reads mapped. Genes of high expression are in red, and low expression in blue. (**B**) Differential expression volcano plot: red dots represent genes that are significantly upregulated, blue dots represent those that are significantly downregulated and grey dots represent those that are not significant. The genes with significant differential expression were defined according to the criteria of |log2FoldChange| > 1 and adjusted *p*-value < 0.05.

For differentially expressed genes (DEGs), the differential levels of gene expression between the mAb MT99/3-treated group and the isotype-matched control mAb-treated group were determined. The DEG clusters were analyzed in a heat map (Figure 3A). In three independent samples of each treated group, DEGs were identified in the same cluster. This indicated a good reproducibility of the treatments. A total of 61,806 differentially expressed genes were identified between the control and mAb MT99/3-treated groups. The DEG statistics indicated 101 upregulated genes and 2 downregulated genes in the mAb MT99/3-treated group compared with the isotype-matched control-treated group (Figure 3B).

### 3.3. Analysis of KEGG Pathway Enrichment of DEGs 

**Figure 4:**
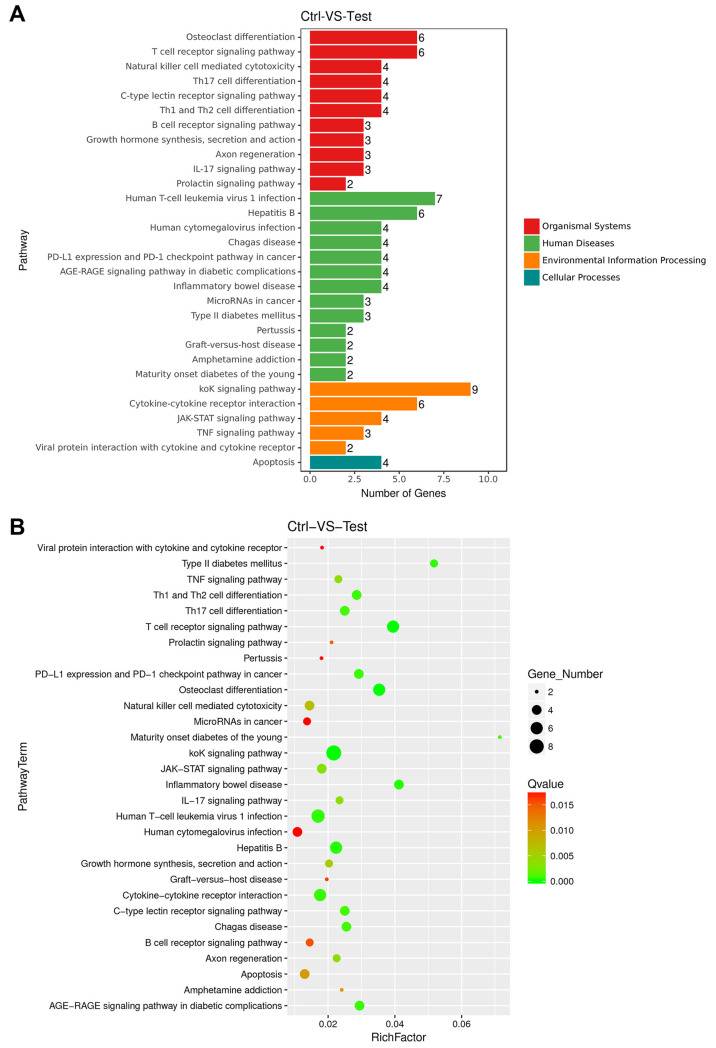
Kyoto Encyclopedia of Genes and Genomes (KEGG) pathways enrichment analyses of DEGs for control versus mAb MT99/3. (**A**) The top 30 most significant KEGG Pathway annotation and classification. (**B**) Scatter plot of the most significant top 30 pathways in KEGG enrichment. Richfactor refers to the ratio of differentially expressed gene numbers annotated in this pathway term to the total gene number annotated in this pathway term. The larger the Richfactor, the greater the degree of enrichment. The size of the dot is positively correlated with the number of differential genes in the pathway term. The Qvalue is the P value after multiple hypothesis testing and ranges between 0 and 1; the closer to zero, the more significant the enrichment. A color code is used to indicate different Qvalue ranges.

KEGG Pathway Enrichment analyses of DEGs in the control-treated group compared with the mAb MT99/3-treated group were performed. The significant DEGs were clustered into four categories of pathways: organismal system, human diseases, environmental information processing and cellular processes (Figure 4A). A bar graph and a scatter plot were used to show the top 30 pathways that were most significantly enriched for KEGG Pathway analysis (Figure 4A,B). In this scatter plot, the degree of KEGG enrichment was measured by Richfactor, Qvalue and the number of genes enriched in this pathway. The entire genes of the most significant top 30 pathways in KEGG enrichment were listed in Supplementary Table S2. Three apoptosis-related signaling pathways, including apoptosis, TNF signaling pathway and JAK-STAT signaling pathway, were significantly enriched in the top 30 pathways. These three pathways might be involved in CD99 signaling upon mAb MT99/3 treatment (Table 1). Moreover, out of the top 30 apoptosis-related signaling pathways, another two were also considered, namely the PI3K-Akt signaling pathway and the NF-kappa B signaling pathway (Table 1). Altogether, the 13 upregulated genes in these pathways may have close relationships to the cell apoptosis in malignant T-ALL cells caused by mAb MT99/3 treatment (Table 1).

### 3.4. DEGs Were Verified by RT-qPCR 

**Figure 5:**
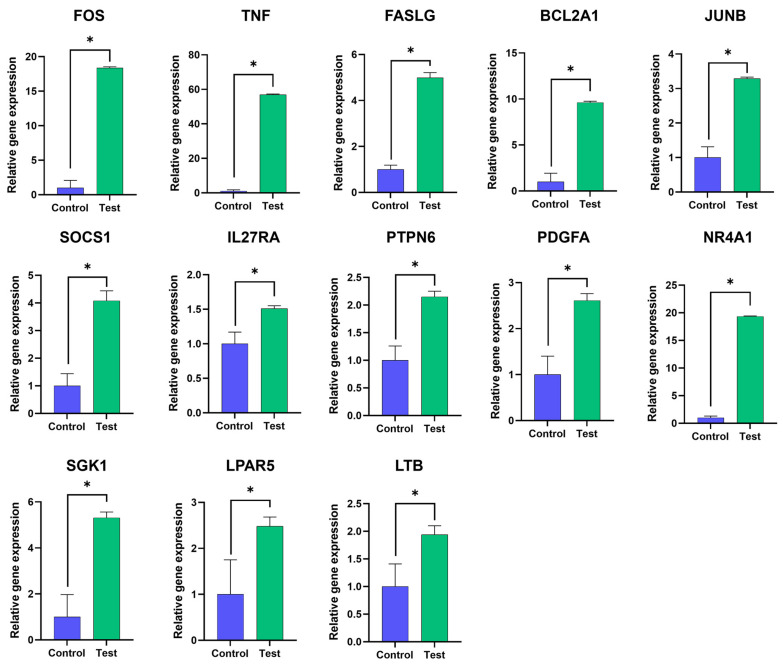
RT-qPCR verification of DEGs. The mRNA expression levels of the 13 DEGs as indicated obtained from the mAb MT99/3 treatment (test) and isotype-matched control mAb treatment (control) of Jurkat E6.1 cells were quantified using RT-qPCR. The housekeeping gene GAPDH was used to normalize the relative gene expression levels. The relative gene expression level was analyzed using the 2^−ΔΔCT^ method. The bar graphs are expressed as mean ± SD of three independent experiments. Mann–Whitney U test was used for comparison, * *p* < 0.05.

To verify the accuracy of the transcriptome sequencing data, gene expression levels of 13 DEGs obtained from RNA-seq data were determined by RT-qPCR. These 13 upregulated genes which might be involved in apoptosis-related signaling pathways, namely FOS, TNF, FASLG, BCL2A1, JUNB, SOCS1, IL27RA, PTPN6, PDGFA, NR4A1, SGK1, LPAR5 and LTB, were analyzed. As shown in Figure 5, all tested genes were significantly upregulated. The results showed a very high correlation between RT-qPCR analysis and RNA-seq data. The results indicated that RNA-seq and transcriptome analyses were reliable.

### 3.5. Identification of CD99 Epitope Recognized by mAb MT99/3

**Figure 6:**
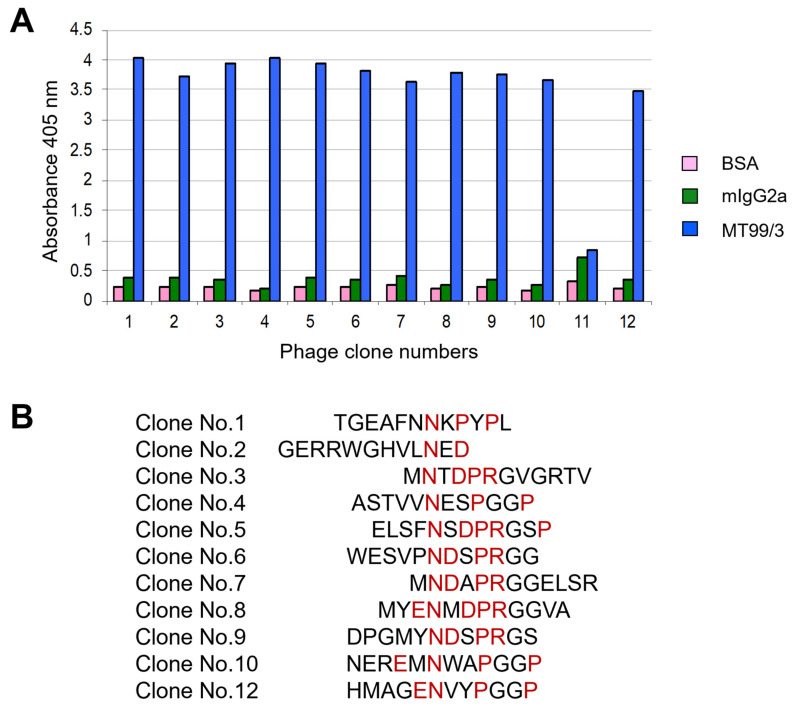
Epitope mapping via phage display random peptide library. (**A**) Binding activity of selected phage clones to mAb MT99/3 via phage peptide ELISA. The ELISA wells were coated with mAb MT99/3 or negative controls, including mIgG2a (isotype-matched control mAb 4G2) and BSA. Bound phage was detected at an absorbance of 405 nm. (**B**) Amino acid sequences of the 12-mers peptides from 11 selected phage clones. The amino acid sequences of CD99 epitope interacting with mAb MT99/3 are predicted and indicated in red letters.

As mAb MT99/3 could induce malignant T cell line apoptosis, we raised the question of whether mAb MT99/3 might trigger a specific epitope on the CD99 molecule. To address this question, the epitope recognized by mAb MT99/3 was determined by bio-panning of the phage-peptide display. The results demonstrated that 11 phage clones showed positive reactivity with mAb MT99/3 (Figure 6A). The DNA and amino acid sequences of the 11 phage clones were determined. Amino acid sequences of the 12-mers peptides from the 11 phage clones are shown in Figure 6B. The CD99 epitope recognized by mAb MT99/3 was, then, predicted using UniProt (P14209-1). The mAb MT99/3 bound to the probable ENDDPRPP at residues 63-70 of CD99 protein. 

**Figure 7:**
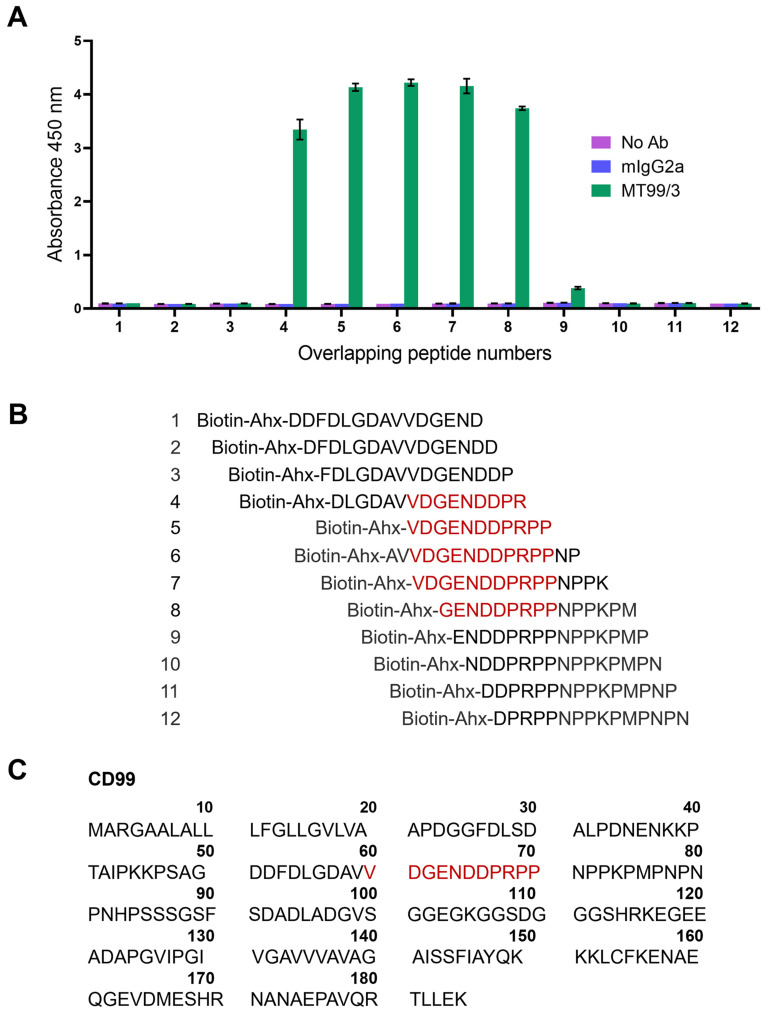
Epitope mapping via overlapping peptide libraries. (**A**) Binding activity of mAb MT99/3 to overlapping peptide libraries of CD99 extracellular domain residues 51–80 by ELISA. The avidin pre-coated ELISA wells were coated with 12 overlapping peptides tagged biotin-6-aminohexanoic acid (Ahx). The mAb MT99/3, mIgG2a (isotype-matched control mAb 4G2), or buffer (No Ab) was added into overlapping peptide libraries. The binding activity of antibodies to peptides was measured at an absorbance of 450 nm. The bar graphs are shown as mean ± SD of two independent experiments. (**B**) Amino acid sequences of 12 CD99 overlapping peptides are exhibited. Red letters indicate the predicted amino acid residues of CD99 epitope recognized by mAb MT99/3. (**C**) The amino acid sequence of CD99 was obtained from UniProt (P14209-1). The CD99 epitope at extracellular part correlated to the result of CD99 overlapping peptide libraries is shown in red letters.

The epitope mapping was further performed to confirm the epitope of mAb MT99/3. The CD99 overlapping peptide libraries were designed from residues 51–80 of the CD99 extracellular domain. By ELISA using overlapping peptides as antigens, mAb MT99/3 showed positive reactivity with peptide numbers 4–8 (Figure 7A), the amino acid sequences of which were shown in Figure 7B. The CD99 epitope recognized by mAb MT99/3 was identified as amino acid VDGENDDPRPP at residues 60–70 of the CD99 molecule (Figure 7C).

## 4. Discussion

*CD99* gene encodes two isoforms of CD99 protein, CD99 long form (CD99LF) and CD99 short form (CD99SF) [21]. These two isoforms are expressed in a cell type-specific manner and regulate distinct CD99 functions [21,22]. CD99LF and CD99SF are differentially expressed in T cells [12]. The early stage of thymocytes and malignant T cell lines co-express CD99LF and CD99SF, whereas the mature peripheral blood T cells express only CD99LF. The antibody ligation of CD99 induced apoptosis in primary T-ALL patient samples [14,23], T-ALL cell lines, immature T cells and B cells but does not affect mature cells [13,22,24]. The ligation of two isoforms of CD99 was required to induce apoptosis, while ligation only on CD99LF was not sufficient to induce apoptosis [12]. In addition, previous studies demonstrated that CD99 engagement by antibodies in distinct cell types activated different downstream signaling [25,26,27]. Some anti-CD99 mAbs failed to induce apoptosis in T-ALL cells but the following anti-CD99 mAbs, which are specific to distinct epitopes, exhibited cytotoxicity by inducing T-ALL cell apoptosis [11]. The mAb DN16, which is mouse IgG1 and binds to residues 32–39 of CD99, induced apoptosis in the Jurkat T-ALL cell line in the presence and absence of a secondary antibody crosslinker [28,29]. The mAb 0662, mouse IgG3, recognizing CD99 residues 88–97, exhibited apoptosis in the Jurkat T-ALL cell line [13,28]. The mAb Ad20, mouse IgM, that binds to CD99 at a distant region, and which was compared with the mAb 0662, could rapidly induce apoptosis in T-ALL cell lines, Jurkat and MOLT-4, but had no effects on the SUPT-1 (T-LBL) cell line and normal T cells [13]. In 2022, human IgG-based tetravalent antibody 10A1 against CD99 residues 63–76 was produced and demonstrated the induction of cell apoptosis in T-ALL patient samples and T-ALL cell lines but not in healthy peripheral blood cells [14]. These reports provided the information that anti-CD99 antibodies might be a potential therapeutic antibody for the treatment of T-ALL with minimal toxicity to normal cells.

In our research center, anti-CD99 mAb, named MT99/3, was generated [15]. In this study, mAb MT99/3 was employed to investigate its property in the induction of cell apoptosis in T-ALL/T-LBL cell lines. The Jurkat E6.1, MOLT-4 and SUPT-1 cell lines were used in representative of T-ALL/T-LBL. In addition to malignant T cells, the effect of mAb MT99/3 was also investigated on normal PBMCs. At first, we confirmed that our generated mAb MT99/3 reacted to CD99 molecules expressed on malignant T cells and PBMCs. As was described, the mAb MT99/3 showed positive reactivity to all T-ALL/T-LBL cell types, as well as PBMCs [11,15,30]. CD99 expression levels on T-ALL cell lines were the strongest [11]. The mAb MT99/3 exerted a killing effect by induction of cell apoptosis in all tested T cell lines. This, however, occurred only in the presence of a secondary antibody crosslinker. It has been reported that the induction of apoptosis in T-ALL required the clustering of a minimum of three CD99 molecules and an antibody valency ≥3 is able to activate cell apoptosis [14]. In agreement with the previous report, our results suggested that mAb MT99/3 also required the cluster of CD99 molecules on malignant T cells for the activation of cell apoptosis. Levels of apoptosis caused by mAb MT99/3 were around 40–70% in T-ALL and around 10–20% in T-LBL, indicating that mAb MT99/3 induced levels of apoptosis in T-ALL higher than T-LBL, which is low CD99 expression. Importantly, this anti-CD99 mAb MT99/3 did not induce apoptosis in non-malignant peripheral blood cells. Anti-CD99 antibodies [13,14], as well as mAb MT99/3, had no cytotoxicity effect on non-malignant peripheral blood cells, probably depending on the different isoform expression and downstream signaling between non-malignant peripheral blood cells and malignant T cells [13,14]. However, so far, the molecular mechanism of CD99 involved in the apoptosis of malignant T cells upon anti-CD99 mAb engagement remains elusive. 

To provide new insight and a comprehensive understanding of molecular mechanisms underlying CD99 ligation, RNA-seq and next-generation transcriptome analysis of T-ALL upon anti-CD99 mAb engagement were performed. The results demonstrated that 61,806 differentially expressed genes were identified between the control and mAb MT99/3-treated groups. The results demonstrated that 101 genes were significantly upregulated, and 2 genes were significantly downregulated in mAb MT99/3 treatment versus control. The 13 upregulation genes, including FOS, TNF, FASLG, BCL2A1, JUNB, SOCS1, IL27RA, PTPN6, PDGFA, NR4A1, SGK1, LPAR5 and LTB, were in pathways that close relationships to the malignant T cell apoptosis caused by mAb MT99/3 treatment. These differentially expressed genes were verified by RT-qPCR. The RT-qPCR results were very similar to RNA-seq data, indicating that the transcriptome analysis results were reliable. To the best of our knowledge, this is the first time it has been demonstrated that the molecular basis of CD99 is involved in the apoptosis of malignant T cells at the transcriptome level. Crosstalk of molecules in several pathways, including apoptosis pathway, TNF signaling pathway, JAK-STAT signaling pathway, PI3K-Akt signaling pathway and NF-kappa B signaling pathway, may be implicated in the apoptosis of CD99 engagement by mAb MT99/3 in malignant T cells. Our finding is related to previous reports [31]. CD99SF engagement by antibodies in breast cancer increased the expression of JunD and FosB AP-1 factors, along with the activation of PI3K-Akt signaling pathway by phosphorylation of Src, Akt, p38 MAPK, ERK and JNK [31]. Moreover, the JNK-AP-1 pathway could enhance the expression of pro-apoptotic genes such as TNF-α and FasL, which contain AP-1-binding sites [32,33]. TNF-α and FasL play an important role in the apoptotic function of many cell types [34,35]. Therefore, the ligation of CD99 by mAb MT99/3 in T-ALL might activate Fos and JunB resulting in the increased expression of TNF-α and FasL, which are key apoptotic molecules and eventually induce Jurkat cell apoptosis.

The ligation of CD99 by mAbs recognizing distinct epitopes has been revealed to activate different death signals [22,28]. Thus, in this study, identification of the epitope recognized by mAb MT99/3 was carried out by phage display random peptide library and overlapping peptide libraries. Surprisingly, it was found that mAb MT99/3 binds to amino acid VDGENDDPRPP residues 60–70 of CD99. This region has not been previously identified and is different from the epitopes of other anti-CD99 mAbs [11,13,14,28,29]. This finding indicates that mAb MT99/3 triggered a new epitope on the CD99 molecule and induced apoptosis-related genes in T-ALL. The information obtained from our study may serve as a new therapeutic target for T-ALL therapy. This contributes to a better understanding of molecular mechanisms of CD99 ligation with antibodies recognizing new epitopes. Additionally, these findings not only provide new insights into CD99 implicated in apoptosis of T-ALL, but also indicate that the anti-CD99 mAb clone MT99/3 may be another candidate antibody for development of therapeutic antibody for T-ALL therapy. 

## 5. Limitations

The limitations of this study are as follows. First, the results were obtained by using representative T-ALL cell lines. Other malignant T cell lines and primary T-ALL samples should be investigated in further study. Second, mAb MT99/3 is a mouse monoclonal antibody; for clinical application, this type of antibody might be inappropriate. Humanized mAb MT99/3 should be constructed to prevent rejection and harm in clinical use. Third, the possible mechanism of the mAb MT99/3 inducing apoptosis in malignant T cells at the transcriptome level was revealed. Future studies should further investigate the underlying mechanisms at the protein level.

## Figures and Tables

**Table 1:** DEGs in apoptosis-related signaling pathways of mAb MT99/3-treated Jurkat T cells.

Pathway Term	Gene ID	Description	Gene Symbol	Control FPKM ^※^	mAb MT99/3 FPKM ^※^	Regulated by mAb MT99/3
Apoptosis	ENSG00000170345	Fos proto-oncogene, AP-1 transcription factor subunit	FOS	0.35	6.16	Up
	ENSG00000232810	Tumor necrosis factor	TNF	0.10	1.59	Up
	ENSG00000117560	Fas ligand	FASLG	0.00	0.56	Up
	ENSG00000140379	BCL2-related protein A1	BCL2A1	0.20	0.95	Up
TNF signaling pathway	ENSG00000171223	JunB proto-oncogene, AP-1 transcription factor subunit	JUNB	21.47	48.43	Up
	ENSG00000170345	Fos proto-oncogene, AP-1 transcription factor subunit	FOS	0.35	6.16	Up
	ENSG00000232810	Tumor necrosis factor	TNF	0.10	1.59	Up
JAK-STAT signaling pathway	ENSG00000185338	Suppressor of cytokine signaling 1	SOCS1	1.19	7.21	Up
	ENSG00000104998	Interleukin 27 receptor subunit alpha	IL27RA	3.18	7.19	Up
	ENSG00000111679	Protein tyrosine phosphatase non-receptor type 6	PTPN6	18.56	49.36	Up
	ENSG00000197461	Platelet-derived growth factor subunit A	PDGFA	1.36	3.13	Up
PI3K-Akt signaling pathway	ENSG00000123358	Nuclear receptor subfamily 4 group A member 1	NR4A1	1.49	23.57	Up
	ENSG00000118515	Serum/glucocorticoid-regulated kinase 1	SGK1	0.72	2.10	Up
	ENSG00000197461	Platelet-derived growth factor subunit A	PDGFA	1.36	3.13	Up
	ENSG00000117560	Fas ligand	FASLG	0.00	0.56	Up
	ENSG00000184574	Lysophosphatidic acid receptor 5	LPAR5	7.28	16.72	Up
NF-kappa B signaling pathway	ENSG00000232810	Tumor necrosis factor	TNF	0.10	1.59	Up
	ENSG00000227507	Lymphotoxin beta	LTB	2.47	5.18	Up
	ENSG00000140379	BCL2-related protein A1	BCL2A1	0.20	0.95	Up

^※^ fragments per kilobase of exon model per million reads mapped.

## Data Availability

Most data generated or analyzed during this study are included in this published article and its Supplementary Materials. The transcriptome data generated for this study can be found in the Sequence Read Archive, accession number PRJNA1020530.

## Supplementary Materials

The following supporting information can be downloaded at: https://www.mdpi.com/article/10.3390/antib13020042/s1, Figure S1: immunofluorescence staining of T cell lines and human PBMCs with mAb MT99/3; Figure S2: antibody-treated Jurkat E6.1 cells for RNA extraction; Table S1: filtered data quality; Table S2: differentially expressed genes of the most significant top 30 pathways in KEGG enrichment.
