# Supplementary material for: Transcriptome Analysis Reveals the Induction of Apoptosis-Related Genes by a Monoclonal Antibody against a New Epitope of CD99 on T-Acute Lymphoblastic Leukemia
Source: Antibodies (Basel). 2024 May 17;13(2):42. doi: 10.3390/antib13020042 (PMC11130895; doi:10.3390/antib13020042)
Supplement: Supplementary file 1 [file antibodies-13-00042-s001.zip › antibodies-2994813-supplementary materials.pdf]

Supplementary materials

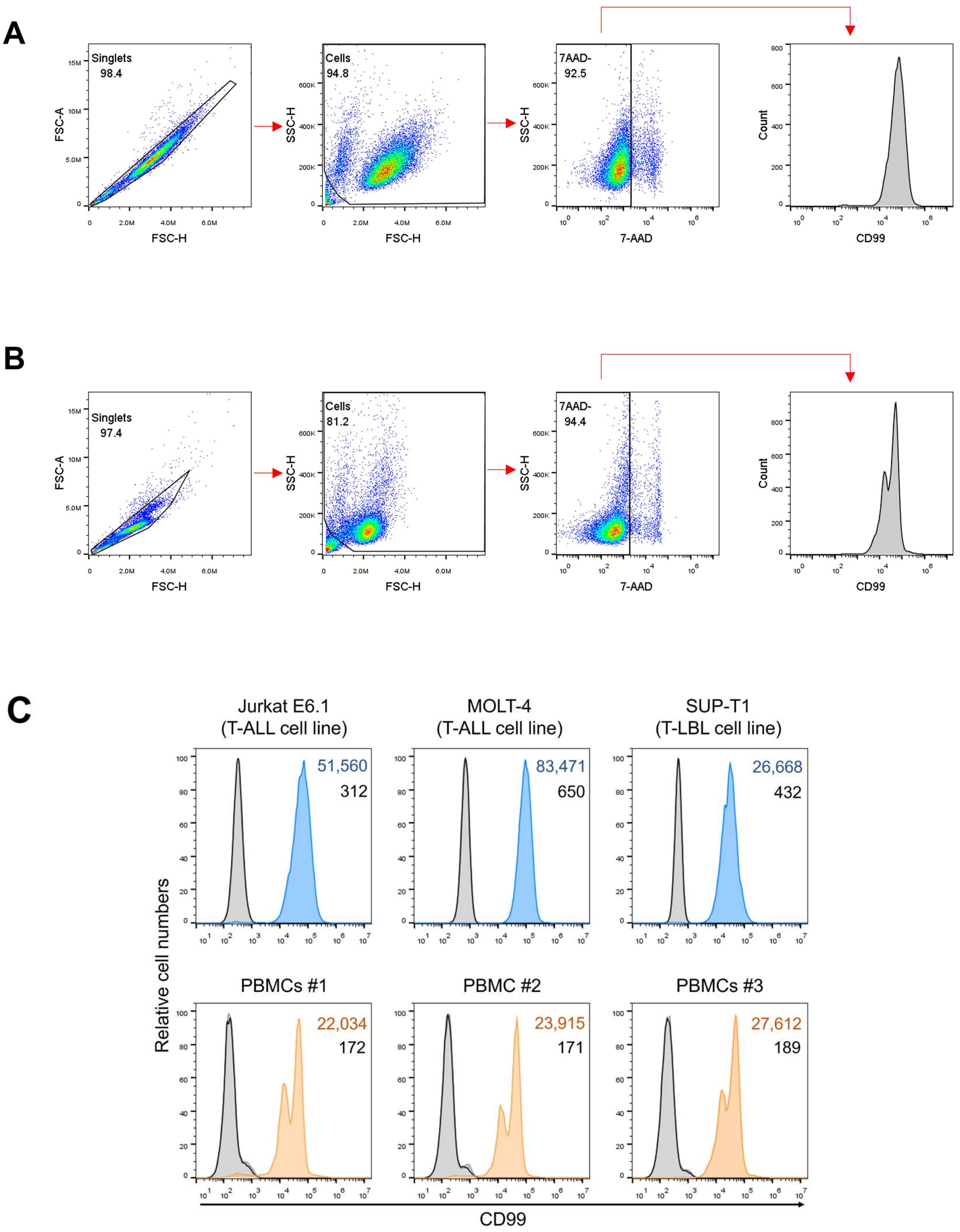

Supplementary Figure S1. Immunofluorescence staining of T cell lines and human PBMCs with mAb MT99/3. T cell lines and human PBMCs (*n*=3) were incubated with 20 µg/mL of mAb MT99/3 (mouse IgG2a) or isotype matched control mAb 4G2 or without antibody for 30 minutes after Fc receptor blocking. Bound antibodies were detected using Alexa Flour-488-anti-mouse IgG Abs (H + L chains specific). After that, the stained cells were suspended in 7-AAD solution and analyzed by flow cytometry. Gating strategies for flow cytometric analysis. The first gating of T cell lines (A) and

PBMCs (B) was the gating of singlet cells in plot between FSC-H and FSC-A. Singlet cells were gated to plot between FSC-H and SSC-H. The cells from the gate of FSC-H and SSC-H were analyzed for 7-AAD-. The lived cells (7-AAD-) were gated to analyze the reactivity of mAb MT99/3 using histogram graph. (C) The histograms exhibit the staining of mAb MT99/3 (blue or orange peaks), isotype-matched control mAb 4G2 (clear peaks), or without mAbs (gray peaks). Geometric mean fluorescence intensity of CD99 expression (colored) and isotype-matched control mAb staining (black) is indicated at the upper right corner of the histogram graph.

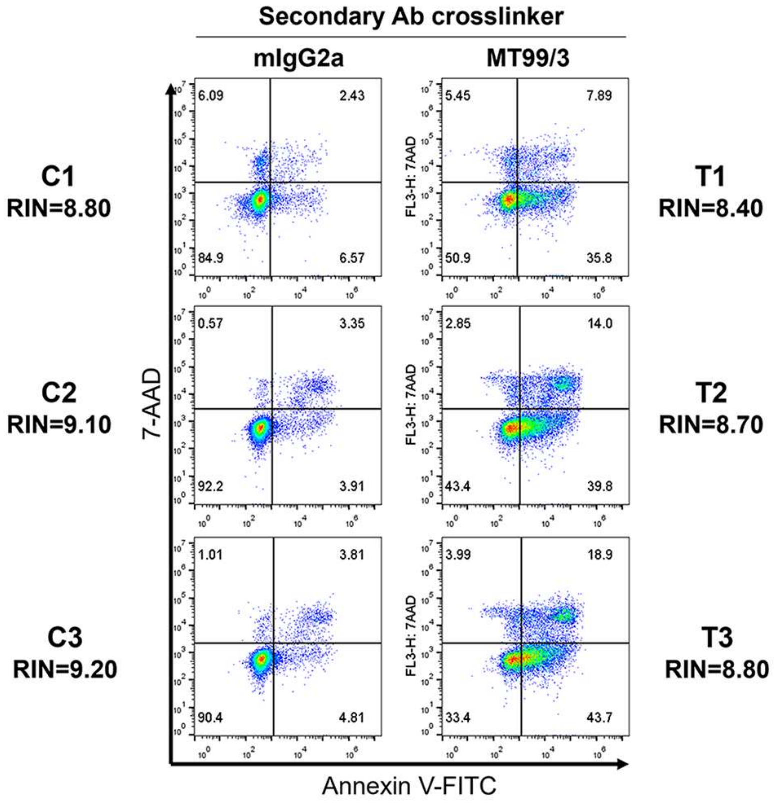

Supplementary Figure S2. Antibody-treated Jurkat E6.1 cells for RNA extraction. Jurkat E6.1 cells were treated with mAb MT99/3 or mIgG2a (isotype-matched control mAb 4G2) in the presence of secondary antibody crosslinker for 90 minutes. Flow cytometric analysis of apoptotic cells was performed using Annexin-V-FITC and 7-AAD before RNA extraction. Three independent experiments were carried out and indicated as T1, T2 and T3 for mAb MT99/3 treatment and C1, C2 and C3 for isotype-matched control mAb treatment. RNA Integrity Number (RIN) after extraction analyzed by Agilent 2100 Bioanalyzer are shown.

Supplementary Table S1. **Filtered data quality**

Sample	Length (bp)	Reads	Bases	Q20 (%)	Q30 (%)	GC (%)
C1	143.52	55,986,084	8,034,966,837	97.54	93.24	48.43
C2	142.79	46,609,370	6,655,449,531	97.86	93.96	48.67
C3	143.45	47,439,424	6,805,317,090	97.49	93.14	48.18
T1	144.05	52,879,358	7,617,517,816	97.65	93.52	48.64
T2	143.10	53,325,982	7,631,041,468	97.58	93.37	48.56
T3	143.43	50,534,894	7,248,391,288	97.42	92.97	48.36

Supplementary Table S2. Differentially expressed genes of the most significant top 30 pathways in KEGG enrichment

Pathway term	genes belong to each pathway	
	Upregulated genes	Downregulated genes
Viral protein interaction with cytokine and cytokine receptor	CXCR3, TNF	/
Type II diabetes mellitus	SOCS1, TNF, MAFA	/
TNF signaling pathway	JUNB, FOS, TNF	/
Th1 and Th2 cell differentiation	NFATC1, FOS, TBX21, MAF	/
Th17 cell differentiation	NFATC1, IL27RA, FOS, TBX21	/
T cell receptor signaling pathway	NFATC1, ICOS, PTPN6, FOS, TNF, PDCD1	/
Prolactin signaling pathway	SOCS1, FOS	/
Pertussis	FOS, TNF	/
PD-L1 expression and PD-1 checkpoint pathway in cancer	NFATC1, PTPN6, FOS, PDCD1	/
Osteoclast differentiation	NFATC1, JUNB, SOCS1, FOS, TNF, FOSL1	/
Natural killer cell mediated cytotoxicity	NFATC1, PTPN6, TNF, FASLG	/
MicroRNAs in cancer	SPRY2, SOCS1, PDGFA	/
Maturity onset diabetes of the young	MAFA, FOXA2	/
koK signaling pathway	DUSP2, NR4A1, DUSP5, NFATC1, PDGFA, FOS, TNF, FASLG, DUSP6	/
JAK-STAT signaling pathway	SOCS1, IL27RA, PTPN6, PDGFA	/
Inflammatory bowel disease	NFATC1, TNF, TBX21, MAF	/

Supplementary Table S2. Differentially expressed genes of the most significant top 30 pathways in KEGG enrichment (cont.)

Pathway term	Symbols of genes belong to each pathway	
	Upregulated genes	Downregulated genes
IL-17 signaling pathway	FOS, TNF, FOSL1	/
Human T-cell leukemia virus 1 infection	ZFP36, EGR2, NFATC1, EGR1, FOS, TNF, FOSL1	/
Human cytomegalovirus infection	NFATC1, PTGER4, TNF, FASLG	/
Hepatitis B	EGR2, NFATC1, EGR3, FOS, TNF, FASLG	/
Growth hormone synthesis, secretion and action	JUNB, SOCS1, FOS	/
Graft-versus-host disease	TNF, FASLG	/
Cytokine-cytokine receptor interaction	IL27RA, CXCR3, TNF, TNFRSF12A, LTB, FASLG	/
C-type lectin receptor signaling pathway	EGR2, NFATC1, EGR3, TNF	/
Chagas disease	FOS, TNF, FASLG, SERPINE1	/
B cell receptor signaling pathway	NFATC1, PTPN6, FOS	/
Axon regeneration	DUSP2, DUSP5, DUSP6	/
Apoptosis	FOS, TNF, FASLG, BCL2A1	/
Amphetamine addiction	FOS, ARC	
AGE-RAGE signaling pathway in diabetic complications	NFATC1, EGR1, TNF, SERPINE1	/
